# Participation in a national diagnostic research study: assessing the patient experience

**DOI:** 10.1186/s13023-023-02695-5

**Published:** 2023-04-10

**Authors:** Lindsay E. Rosenfeld, Kimberly LeBlanc, Anna Nagy, Braeden K. Ego, The Undiagnosed Diseases Network, Maria T. Acosta, Margaret Adam, David R. Adams, Raquel L. Alvarez, Justin Alvey, Laura Amendola, Ashley Andrews, Euan A. Ashley, Carlos A. Bacino, Guney Bademci, Ashok Balasubramanyam, Dustin Baldridge, Jim Bale, Michael Bamshad, Deborah Barbouth, Pinar Bayrak-Toydemir, Anita Beck, Alan H. Beggs, Edward Behrens, Gill Bejerano, Hugo J. Bellen, Jimmy Bennett, Beverly Berg-Rood, Jonathan A. Bernstein, Gerard T. Berry, Anna Bican, Stephanie Bivona, Elizabeth Blue, John Bohnsack, Devon Bonner, Lorenzo Botto, Brenna Boyd, Lauren C. Briere, Elly Brokamp, Gabrielle Brown, Elizabeth A. Burke, Lindsay C. Burrage, Manish J. Butte, Peter Byers, William E. Byrd, John Carey, Olveen Carrasquillo, Thomas Cassini, Ta Chen Peter Chang, Sirisak Chanprasert, Hsiao-Tuan Chao, Gary D. Clark, Terra R. Coakley, Laurel A. Cobban, Joy D. Cogan, Matthew Coggins, F. Sessions Cole, Heather A. Colley, Cynthia M. Cooper, Heidi Cope, Rosario Corona, William J. Craigen, Andrew B. Crouse, Michael Cunningham, Precilla D’Souza, Hongzheng Dai, Surendra Dasari, Joie Davis, Jyoti G. Dayal, Esteban C. Dell’Angelica, Katrina Dipple, Daniel Doherty, Naghmeh Dorrani, Argenia L. Doss, Emilie D. Douine, Laura Duncan, Dawn Earl, David J. Eckstein, Lisa T. Emrick, Christine M. Eng, Marni Falk, Elizabeth L. Fieg, Paul G. Fisher, Brent L. Fogel, Irman Forghani, William A. Gahl, Ian Glass, Bernadette Gochuico, Page C. Goddard, Rena A. Godfrey, Katie Golden-Grant, Alana Grajewski, Don Hadley, Sihoun Hahn, Meghan C. Halley, Rizwan Hamid, Kelly Hassey, Nichole Hayes, Frances High, Anne Hing, Fuki M. Hisama, Ingrid A. Holm, Jason Hom, Martha Horike-Pyne, Alden Huang, Sarah Hutchison, Wendy Introne, Rosario Isasi, Kosuke Izumi, Fariha Jamal, Gail P. Jarvik, Jeffrey Jarvik, Suman Jayadev, Orpa Jean-Marie, Vaidehi Jobanputra, Lefkothea Karaviti, Jennifer Kennedy, Shamika Ketkar, Dana Kiley, Gonench Kilich, Shilpa N. Kobren, Isaac S. Kohane, Jennefer N. Kohler, Susan Korrick, Mary Kozuira, Deborah Krakow, Donna M. Krasnewich, Elijah Kravets, Seema R. Lalani, Byron Lam, Christina Lam, Brendan C. Lanpher, Ian R. Lanza, Brendan H. Lee, Roy Levitt, Richard A. Lewis, Pengfei Liu, Xue Zhong Liu, Nicola Longo, Sandra K. Loo, Joseph Loscalzo, Richard L. Maas, Ellen F. Macnamara, Calum A. MacRae, Valerie V. Maduro, Rachel Mahoney, May Christine V. Malicdan, Laura A. Mamounas, Teri A. Manolio, Rong Mao, Kenneth Maravilla, Ronit Marom, Gabor Marth, Beth A. Martin, Martin G. Martin, Julian A. Martínez-Agosto, Shruti Marwaha, Jacob McCauley, Allyn McConkie-Rosell, Elisabeth McGee, Heather Mefford, J. Lawrence Merritt, Matthew Might, Ghayda Mirzaa, Eva Morava, Paolo Moretti, John Mulvihill, Mariko Nakano-Okuno, Stanley F. Nelson, John H. Newman, Sarah K. Nicholas, Deborah Nickerson, Shirley Nieves-Rodriguez, Donna Novacic, Devin Oglesbee, James P. Orengo, Laura Pace, Stephen Pak, J. Carl Pallais, Christina G. S. Palmer, Jeanette C. Papp, Neil H. Parker, John A. Phillips, Jennifer E. Posey, Lorraine Potocki, Barbara N. Pusey Swerdzewski, Aaron Quinlan, Deepak A. Rao, Anna Raper, Wendy Raskind, Genecee Renteria, Chloe M. Reuter, Lynette Rives, Amy K. Robertson, Lance H. Rodan, Jill A. Rosenfeld, Natalie Rosenwasser, Francis Rossignol, Maura Ruzhnikov, Ralph Sacco, Jacinda B. Sampson, Mario Saporta, Judy Schaechter, Timothy Schedl, Kelly Schoch, Daryl A. Scott, C. Ron Scott, Vandana Shashi, Jimann Shin, Edwin K. Silverman, Janet S. Sinsheimer, Kathy Sisco, Edward C. Smith, Kevin S. Smith, Emily Solem, Lilianna Solnica-Krezel, Ben Solomon, Rebecca C. Spillmann, Joan M. Stoler, Kathleen Sullivan, Jennifer A. Sullivan, Angela Sun, Shirley Sutton, David A. Sweetser, Virginia Sybert, Holly K. Tabor, Queenie K.-G. Tan, Amelia L. M. Tan, Mustafa Tekin, Fred Telischi, Willa Thorson, Cynthia J. Tifft, Camilo Toro, Alyssa A. Tran, Rachel A. Ungar, Tiina K. Urv, Adeline Vanderver, Matt Velinder, Dave Viskochil, Tiphanie P. Vogel, Colleen E. Wahl, Melissa Walker, Stephanie Wallace, Nicole M. Walley, Jennifer Wambach, Jijun Wan, Lee-kai Wang, Michael F. Wangler, Patricia A. Ward, Daniel Wegner, Monika Weisz Hubshman, Mark Wener, Tara Wenger, Monte Westerfield, Matthew T. Wheeler, Jordan Whitlock, Lynne A. Wolfe, Kim Worley, Changrui Xiao, Shinya Yamamoto, John Yang, Zhe Zhang, Stephan Zuchner, Alexa T. McCray

**Affiliations:** 1 Department of Biomedical Informatics, Harvard Medical School, 10 Shattuck Street, Boston, MA 02115 USA; 2 Heller School for Social Policy and Management, Institute for Child, Youth, and Family Policy, Brandeis University, 415 South St., Waltham, MA 02453 USA; 3 Department of Genetics, Stanford University School of Medicine, 291 Campus Drive, Stanford, CA 94305 USA; 4 Division of Clinical Informatics, Beth Israel Deaconess Medical Center, 330 Brookline Ave, Boston, MA 02215 USA

**Keywords:** Patient experience, Rare disease, Undiagnosed disease, Clinical evaluation, Multidisciplinary research, Qualitative methods, Patient perspective

## Abstract

**Introduction:**

The Undiagnosed Diseases Network (UDN), a clinical research study funded by the National Institutes of Health, aims to provide answers for patients with undiagnosed conditions and generate knowledge about underlying disease mechanisms. UDN evaluations involve collaboration between clinicians and researchers and go beyond what is possible in clinical settings. While medical and research outcomes of UDN evaluations have been explored, this is the first formal assessment of the patient and caregiver experience.

**Methods:**

We invited UDN participants and caregivers to participate in focus groups via email, newsletter, and a private participant Facebook group. We developed focus group questions based on research team expertise, literature focused on patients with rare and undiagnosed conditions, and UDN participant and family member feedback. In March 2021, we conducted, recorded, and transcribed four 60-min focus groups via Zoom. Transcripts were evaluated using a thematic analysis approach.

**Results:**

The adult undiagnosed focus group described the UDN evaluation as validating and an avenue for access to medical providers. They also noted that the experience impacted professional choices and helped them rely on others for support. The adult diagnosed focus group described the healthcare system as not set up for rare disease. In the pediatric undiagnosed focus group, caregivers discussed a continued desire for information and gratitude for the UDN evaluation. They also described an ability to rule out information and coming to terms with not having answers. The pediatric diagnosed focus group discussed how the experience helped them focus on management and improved communication. Across focus groups, adults (undiagnosed/diagnosed) noted the comprehensiveness of the evaluation. Undiagnosed focus groups (adult/pediatric) discussed a desire for ongoing communication and care with the UDN. Diagnosed focus groups (adult/pediatric) highlighted the importance of the diagnosis they received in the UDN. The majority of the focus groups noted a positive future orientation after participation.

**Conclusion:**

Our findings are consistent with prior literature focused on the patient experience of rare and undiagnosed conditions and highlight benefits from comprehensive evaluations, regardless of whether a diagnosis is obtained. Focus group themes also suggest areas for improvement and future research related to the diagnostic odyssey.

## Background

The Undiagnosed Diseases Network (UDN) is a large collaborative national research network that aims to help individuals with unknown conditions reach a diagnosis. As such, the UDN has a dual clinical and research mission. Its goals are to provide answers for patients with undiagnosed conditions while also generating new knowledge about underlying mechanisms and human physiology using advanced clinical and genomic technologies [1–3]. The UDN is based in the United States and is funded by the National Institutes of Health (NIH), an entity of the federal government. The UDN has several components: a coordinating center, 12 clinical sites, and 4 core laboratories—a sequencing core that provides exome and genome sequencing for the UDN, a model organisms screening center that investigates the potential pathogenicity of variants identified in the genomes of UDN participants, a metabolomics core that studies the underlying biochemistry of UDN biological samples, and a central biorepository that stores UDN biological samples for future use.

The UDN research study began enrolling participants in 2015, and since then has accepted over 2000 individuals. Accepted participants have conditions that defy diagnosis, often despite many years of engagement with the health care system. Once accepted, each participant undergoes an extensive evaluation at one of the UDN clinical sites. The evaluation involves a comprehensive medical and family history, physical examination, laboratory testing, imaging studies, and biological specimen collection from the participant as well as family members, whenever possible. The UDN research study is highly multidisciplinary. Evaluations involve collaboration among clinicians representing many different specialties, and in the search for a diagnosis, clinicians work closely with scientists based at the core laboratories [4, 5]. The PEER (Participant Engagement and Empowerment Resource) group, consisting of UDN patients and family member representatives, works with UDN researchers to improve the participant experience and inform the broader community about the UDN [6].

For individuals with undiagnosed conditions, uncovering answers related to the underlying cause of symptoms can involve a “diagnostic odyssey” of years of medical evaluation and intervention [7–11]. Genomic testing has begun to be incorporated in routine clinical care to help end these odysseys [12–14], and the medical and psychological impacts of such testing on patients and their families are just beginning to be understood. Recent studies have examined how individuals and parents of children might be affected by genomic testing results. For example, parents or caregivers may be able to cope better with their child’s condition, resulting in an improved quality of life; the results may allow the family to better prepare for the future; the information may provide closure for a diagnostic odyssey; or it may improve access to health care services and open possibilities for targeted treatments [15–17]. Other recent studies focus on the importance of genetic counseling in helping individuals prepare for genomic testing, interpret test results, and manage the emotional impact of receiving, or not receiving, a diagnosis or definitive answer [18–20].

The UDN methods, which include not only genomic testing, but also model organism and metabolomics research, and, importantly, comprehensive multidisciplinary and collaborative clinical evaluations, have enabled its successes and have resulted in diagnosis for more than one third of these extremely difficult UDN cases [2]. UDN methods go well beyond what is possible in most clinical settings, and while the medical and research outcomes of the UDN model have been documented [21–24], little work has been done to formally assess the patient experience. Thus, the objective of the current study is to gain a deeper understanding of the patient and caregiver perspective on participating in the UDN, whether this resulted in a diagnosis or not.

In the following, we report the findings from a qualitative study with UDN participants and caregivers who completed the UDN evaluation. We conducted four focus group sessions with the goal of assessing the experience of participating in the UDN. We identified themes that arose within and across each of the focus groups—(1) adults who didn’t receive a diagnosis (adult undiagnosed), (2) adults who received a diagnosis (adult diagnosed), (3) caregivers of children who did not receive a diagnosis (pediatric undiagnosed), and (4) caregivers of children who received a diagnosis (pediatric diagnosed). We illustrate our analyses and conclusions with quotes from members of the focus groups. We conclude with a discussion of the implications of our study for a broader understanding of the impact of genomic testing and clinical research evaluation on patients and caregivers.

## Methods

### Setting

We conducted a qualitative study using focus groups to understand the experience of diverse (e.g., age, income, gender, race, diagnosis, site) UDN participants. We used thematic analysis, specifically content analysis and a grounded theory approach, to meet the study objective, assessing the experience of participating in the UDN. We followed the Consolidated Criteria for Reporting Quality Research guidelines (COREQ) for best practice reporting of qualitative results [25]. We applied plain language communication principles in preparation of all written documents (e.g., recruitment emails, consent documents), the focus group facilitator guide, as well as for slides and in conversation [26]. Our research team included two genetic counselors, a genetic counseling student, a social epidemiologist, and a biomedical informatician. An extension of this core research team was the UDN PEER [6].

A social epidemiologist (LER) conducted the focus groups with two UDN genetic counselors (KL, AN). LER holds a doctorate in public health with scientist and instructor positions at various academic institutions in her fields of expertise, including qualitative methods, health equity, and disability. LER did not have a prior relationship with the members of the focus groups. She introduced herself as a study team member who also had a personal connection to the work. Prior to Institutional Review Board (IRB) submission, UDN PEER stakeholders engaged in a “lived experience” check to ensure that data collection plans and focus group guides suited participant reality. Four focus groups were used to gather information; collected data were analyzed using thematic analysis. The study protocol was approved by the NIH IRB (protocol 15-HG-0130).

### Recruitment: sample, eligibility criteria, & focus group selection

A genetic counselor on the study team invited all eligible UDN participants and caregivers to participate via an email. We also recruited through posts on the UDN PEER quarterly newsletter and a private participant Facebook group.

Eligible to participate were UDN participants or caregivers of participants who completed their UDN evaluation at least one year prior to the study, were at least 18 years of age, and reported being comfortable speaking English.

We asked individuals interested in being part of the study to complete a survey to assess eligibility. The survey was available through REDCap and collected information about eligibility criteria (e.g., date of evaluation, age, comfort speaking English) and gender, race, ethnicity, household income, contact information, and general schedule availability. We received 237 unique responses to the survey from UDN participants or caregivers. We used self-reported diagnosis as a measure of diagnosis status. We contacted ten individuals from each cohort to schedule a focus group. Alternates were chosen and contacted if an individual was unable to join at the scheduled time. We selected these individuals to represent a cross-section of the aforementioned characteristics, diagnosis, and age of the UDN participant. Focus group members were offered a $50 Amazon gift card as a thank you for their time.

### Data collection: focus groups

A genetic counselor on the team sent selected UDN participants and caregivers a consent document via email with details about the study. Each focus group member assented by signing on to Zoom and participating in the focus group.

We designed a focus group guide to gather the perspectives of the UDN experience from participants and caregivers. We developed focus group questions based on research team expertise, research literature focused on patients with rare and undiagnosed conditions, and feedback from UDN PEER. The guide included questions and prompts for each of five themes: (1) what participants got out of the UDN evaluation, and the impact of the UDN experience on (2) medical care, (3) health or condition, (4) interactions with others, and (5) outlook.

We conducted four focus groups: (1) adult undiagnosed, (2) adult diagnosed, (3) pediatric undiagnosed, and 4) pediatric diagnosed. We created focus groups based on age and diagnostic status; these characteristics have been previously shown to impact patient experiences of the UDN evaluation [27].

We held focus groups via Zoom from March 24 to March 31, 2021. Discussions with two colleagues, experts in focus group facilitation both prior to and during the COVID-19 pandemic, guided virtual focus group logistics. Key recommendations, which were all implemented, included limiting time to ≤ 60 min total (including introductory conversation), and participation of 8–10 people. We aimed for a minimum of five members per focus group, which has been shown to be the fewest number needed for response reliability [28].

We recorded all focus groups and used auto-transcription via Zoom. Three research team members were present—LER facilitated each focus group, KL helped ensure all raised hands were recognized, and AN focused on logistics, including letting people into the private Zoom space and handling technical issues. All three team members took notes and field notes were jointly discussed and compiled immediately after focus group sessions.

BKE listened to each audio recording to ensure that auto-transcription matched the audio, correcting errors as needed. During this process, BKE removed all identifying information and assigned ID numbers to each of the members. Finally, a random check of transcripts (KL) confirmed transcription matched audio.

### Analysis

We used content analysis and a grounded theory approach to guide thematic analysis of focus group transcripts. This approach focuses on the ideas arising from the data, rather than exploring data for previously conceived themes [29]. Content analysis allows for systematic classification of textual data to reach an understanding of the text by uncovering themes and patterns in the data. Focus groups, which allowed participant discussion by diagnosis and age cohort, were most appropriate for elucidating the impact of participation on patients and families, beyond the number of specialist visits or diagnoses [30]. As part of this analysis process, two coders (KL, BKE) independently reviewed each transcript, identified relevant blocks of text, and created codes that captured the main idea of each quote. The two coders conferred to reach consensus on text and codes. Following code consensus, they independently assigned themes to each code and again conferred to reach consensus on themes. These coders then met with a third coder (LER) to review codes and themes for each transcript. The three coders reached consensus for all codes and themes and used Microsoft Excel to manage the data.

After thematic analysis was complete for all transcripts, the coders reviewed themes within focus groups by question and overall, and then across focus groups by question and overall, conferring on thematic patterns revealed [31]. Finally, the coders brought themes to the broader research team to affirm face validity and to discuss data saturation and themes. This approach is akin to qualitative analytical triangulation techniques in which multiple researchers produce similar interpretations of the data [32]. Themes discussed below are those that highlight a topic that 50% or more of the focus group(s) discussed.

## Results

### Focus group demographics

Thirty individuals participated in focus groups (adult undiagnosed (n = 6), adult diagnosed (n = 7), pediatric undiagnosed (n = 8), pediatric diagnosed (n = 9)). Self-reported demographic characteristics are presented in Table 1. Across focus groups, 66.7% (n = 20) identified as female, 33.3% (n = 10) identified as male. The majority identified as White (56.7%, n = 17) and non-Hispanic/Latinx (80.0%, n = 24); 16.7% (n = 5) identified as Asian, 13.3% (n = 4) as Black or African American, 3.3% (n = 1) as Black or African American and Other (not specified), and 3.3% (n = 1) as American Indian or Alaska Native. In terms of household income, 33.3% (n = 10) reported < $20,000, 3.3% (n = 1) reported $20,000–$39,000, 6.7% (n = 2) reported $40,000–$59,000, 3.3% (n = 1) reported $60,000–$79,000, 20.0% (n = 6) reported > $80,000, and 33.3% (n = 10) selected “prefer not to answer.”

**Table 1:** Focus group demographics

Demographics	Total (N = 30)	Adult undiagnosed (n = 6)	Adult diagnosed (n = 7)	Pediatric undiagnosed (n = 8)	Pediatric diagnosed (n = 9)
*Current gender identity*, %, (n)					
Female	66.7% (20)	83.3% (5)	57.1% (4)	62.5% (5)	66.7% (6)
Male	33.3% (10)	16.7% (1)	42.9% (3)	37.5% (3)	33.3% (3)
*Race*, %, (n)					
White	56.7% (17)	50.0% (3)	71.4% (5)	62.5% (5)	44.4% (4)
Asian	16.7% (5)	33.3% (2)	0% (0)	12.5% (1)	22.2% (2)
Black or African American	13.3% (4)	0% (0)	14.3% (1)	12.5% (1)	22.2% (2)
Black or African American and other (not specified)	3.3% (1)	0% (0)	0% (0)	0% (0)	11.1% (1)
American Indian or Alaska Native	3.3% (1)	0% (0)	14.3% (1)	0% (0)	0% (0)
Race not provided	6.7% (2)	16.7% (1)	0% (0)	12.5% (1)	0% (0)
*Ethnicity*, %, (n)					
Not Hispanic/Latinx	80.0% (24)	83.3% (5)	85.7% (6)	75.0% (6)	77.8% (7)
Hispanic/Latinx	10.0% (3)	0.0% (0)	14.3% (1)	12.5% (1)	11.1% (1)
Unsure/unknown	10.0% (3)	16.7% (1)	0.0% (0)	12.5% (1)	11.1% (1)
*Household income*, %, (n)					
< $20,000	33.3% (10)	66.7% (4)	14.3% (1)	12.5% (1)	44.4% (4)
$20,000–$39,000	3.3% (1)	0.0% (0)	0.0% (0)	12.5% (1)	0.0% (0)
$40,000–$59,000	6.7% (2)	0.0% (0)	14.3% (1)	12.5% (1)	0.0% (0)
$60,000–$79,000	3.3% (1)	0.0% (0)	14.3% (1)	0.0% (0)	0.0% (0)
> $80,000	20.0% (6)	33.3% (2)	0.0% (0)	37.5% (3)	11.1% (1)
Prefer not to answer	33.3% (10)	0.0% (0)	57.1% (4)	25.0% (2)	44.4% (4)

**Fig. 1:**
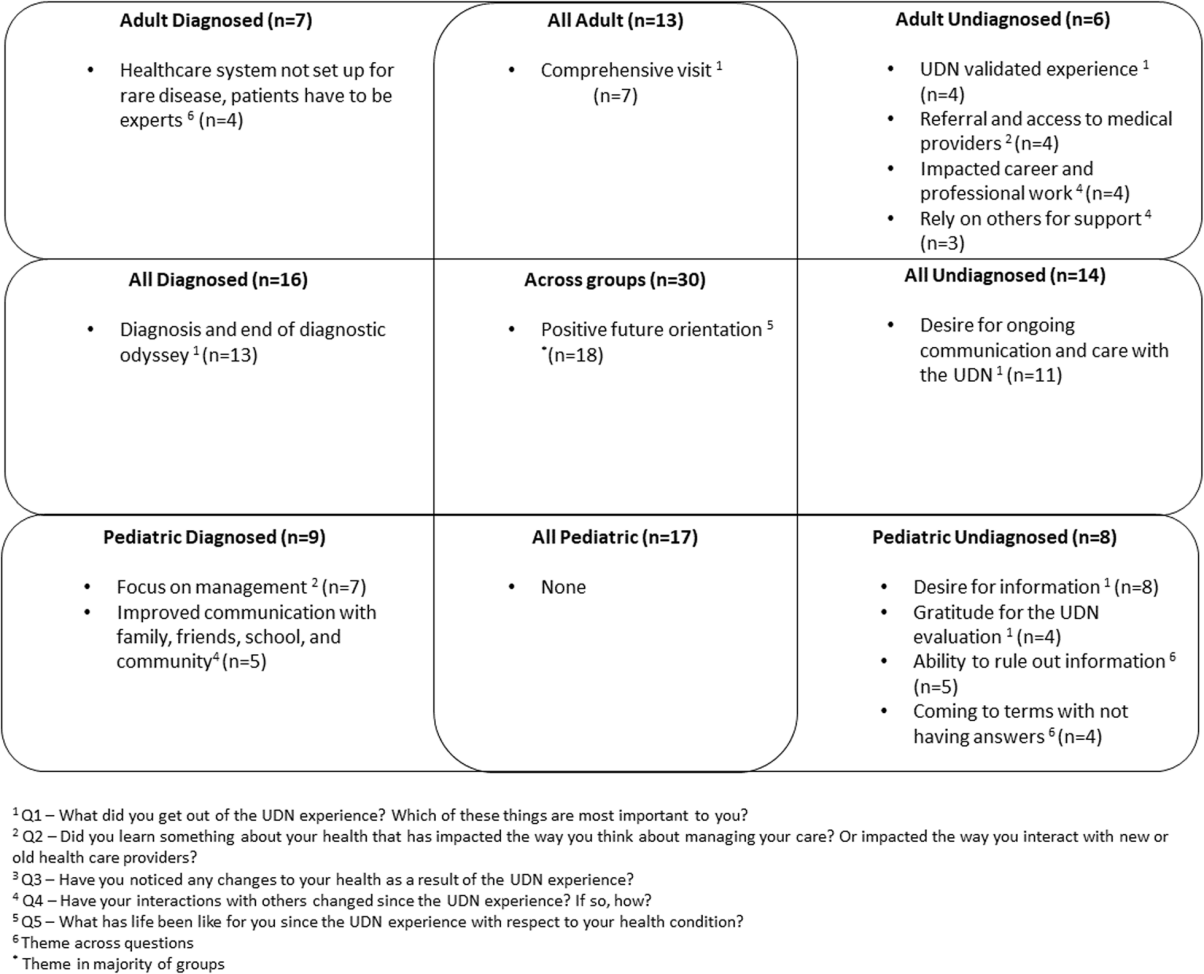
Focus group themes

Themes identified within and across focus groups are presented in Fig. 1.

### Themes within focus groups

*Adult undiagnosed (n* = *6)* Within question 1 (what did you get out of the UDN experience?), focus group members described the UDN evaluation as validating their undiagnosed journey (n = 4). For example, one focus group member noted:

*I imagine other people feel this way too so that when you go to the doctor, and you say “oh, by the way, I have these genetic mutations,” they don't look at you like you're crazy and making up your symptoms. They say, “oh wow,” and then when you say, “I'm in the rare Undiagnosed Diseases study,” they don't just say, “oh, you're making this stuff up. You're crazy.” They say, “oh, wow, that's really cool. I better pay attention.” So as an advocacy tool, that's been the best part of it for me.* (ID 12)

Within question 2 (impact of UDN experience on managing medical care), this focus group discussed the opportunity for referral and access to relevant medical providers following the UDN evaluation (n = 4). Within question 4 (impact of UDN experience on interactions with others), they discussed how the UDN experience impacted career and professional work (n = 4) and helped them to rely more on others for support (n = 3). There were no themes found across questions in this focus group.

*Adult diagnosed (n* = *7)* There were no themes identified within questions in this adult diagnosed focus group. However, there was a theme found across questions about how the healthcare system is not set up for individuals with rare conditions, therefore patients must be the experts on their own condition (n = 4). The following quote from a member of this focus group speaks to this concept:

*I mean we're really like our own doctors, we know our symptoms. They can't tell us no you're not feeling this way. Yes, I'm feeling this way.* (ID 3)

*Pediatric undiagnosed (n* = *8)* Within question 1 (what did you get out of the UDN experience?), pediatric undiagnosed focus group members discussed a continued desire for information (n = 8) and gratitude for the UDN evaluation (n = 4). Across all questions in this focus group, there were two themes about how the UDN experience provided them the ability to 1) rule out information and focus on what was most relevant (n = 5) and 2) come to terms with not having answers (n = 4). For example, one person commented on the gratitude that came with being able to rule out potential syndromes:

*I am so grateful that so many syndromes were ruled out by the UDN visit. And that other symptoms that he was having were not what we thought so I'm glad that even though we don't have an answer, I'm glad that some of the other suspected syndromes were not the case for my [child].* (ID 25)

Another person described how they are coming to terms with not having answers:

*I did have to get into a mind space of we may never know, and not knowing may be what we live with for the rest of his life, um we might not know.* (ID 29)

*Pediatric diagnosed (n* = *9)* Within question 2 (impact of UDN experience on managing medical care), the pediatric diagnosed focus group discussed how the experience helped to focus them on care management, with new strategies for prioritization (n = 7). Within question 4 (impact of UDN experience on interactions with others), this focus group described an improvement in communication with family, friends, school, and community (n = 5). For example, one individual stated:

*And it’s often hard when something is wrong internally but externally he looks perfectly fine. So it's definitely changed the way that everyone around him interacts with him, because we now understand that even externally he looks fine but internally he’s not. And that's been, I guess for him, it's definitely been an improvement in the way he's treated, how he's interacted, it's given us a new level of, like I said, empathy and sympathy.* (ID 20)

### Themes across focus groups

*Adult (undiagnosed and diagnosed) (n* = *13)* Across adult focus groups, discussion about the comprehensiveness of the UDN evaluation arose (n = 7). For example, one member described the experience as follows:

*I think what I got most out of it was that someone was willing to listen to all the many varied health issues that I have. Every time I went to a doctor or a hospital, they were experts in their own field and didn't want to hear about anything else. It's like, “just talk to me about your cardio issues. I don't really care about anything else.” And at UDN was the first time that someone said, I want to hear all of it. And that to me was so enlightening. I got to go to UDN a few years ago. So I'm in my mid-to-late 50s, so it was just so refreshing for my entire life never having had that.* (ID 6)

*Pediatric (undiagnosed and diagnosed) (n* = *17)* No common themes were identified across the pediatric focus groups.

*Undiagnosed (adult and pediatric) (n* = *14)* Across question 1 (what did you get out of the UDN experience?), both the adult and pediatric undiagnosed focus groups discussed a strong desire for ongoing communication and care with the UDN (n = 11). The following describes the thoughts of a caregiver of a pediatric undiagnosed participant:

*But I would just like for somebody from the UDN to tell me “hey, we're still looking at this, you know we haven't given up. We haven't abandoned you. This is still being looked at.” You know, even just every so often, you know, even yearly just to tell us, and remind us that hey we're still looking into this.* (ID 29)

*Diagnosed (adult and pediatric) (n* = *16)* Across question 1 (what did you get out of the UDN experience?), both the adult and pediatric diagnosed focus groups discussed the importance of the diagnosis they were given as part of the UDN evaluation and the diagnosis being the end of a long diagnostic journey (n = 13). One caregiver explained:

*I wasn’t so hopeful at the beginning because I had been through the process with other physicians multiple times and still no answers. So when they came back and they told us that, after you know whole exome and whole genome sequencing and just a bunch of bloodwork and testing and stuff, finding a diagnosis. I literally just broke down in tears just because we finally had an answer from the time he was born we had been wanting an answer and desiring an answer. And we finally had an answer.* (ID 17)

*All focus groups (n* = *30)* Within question 5 (impact of UDN experience on outlook), three of four focus groups highlighted how participating in the UDN evaluation led to their having a positive future orientation (n = 18). Example quotes are highlighted below.

*So I think that it's given me a lot of energy and perseverance and I've had so many setbacks, but I'm not going to give up yet.* (ID 13, adult undiagnosed)

*I’d say that things are easier now than they were the first time that we met with the UDN. I think that part of that is that the UDN has been one of the tools in our toolbox for our kid and her medical stuff.* (ID 30, pediatric undiagnosed)

*I know that we have a lot of questions for her future but as a family, we decided to live more in the present for, for her.* (ID 14, pediatric diagnosed)

## Discussion

The UDN aims to provide answers for patients with unknown conditions. It uses a multidisciplinary clinical research approach involving clinicians from various specialties and researchers who apply cutting edge science, including genomic testing [1, 2]. While UDN medical and research outcomes have been studied [21–24], little work has focused on the patient experience. Our goal in this study was to better understand the patient and caregiver perspective on participating in the UDN, a multi-site network that provides evaluations for some of the most difficult undiagnosed adult and pediatric cases.

Around the world, adult and pediatric patients with rare and complex medical conditions describe delays in diagnosis, misdiagnosis, a lack of care coordination, inexperience among health care providers, and problems with transition from pediatric to adult care [9, 33–38]. Through focus groups, we gained insights from patients and caregivers impacted by rare and undiagnosed conditions regarding these common challenges that can inform improvements in diagnosis and care.

We found that comprehensive multidisciplinary evaluations were useful and tolerable to patients in their diagnostic journeys. In fact, thorough evaluations were important regardless of whether patients received a diagnosis. For example, the majority of focus groups expressed that the UDN evaluation resulted in a positive future orientation. In addition, the adult diagnosed and undiagnosed focus groups valued, in contrast to other adult settings, that the UDN evaluation was uniquely comprehensive and interdisciplinary.

Even though we learned that the evaluation was useful regardless of diagnostic status, it was also apparent that the diagnosis itself was valuable. This was true regardless of available treatment options and even if it meant receiving unwelcome (typically perceived as negative) new information. Adult and pediatric diagnosed focus groups were clear about the significance of receiving a diagnosis through the UDN. Based on prior research, we know the diagnostic odyssey is often a difficult, uncertain time and most are hopeful for a diagnosis as opposed to living in a state of unknown [7, 39–42].

For those without a diagnosis, the UDN evaluation had positive effects as well. For example, the adult undiagnosed focus group highlighted that the experience was validating and helped facilitate future medical care, e.g., referrals to medical providers beyond those just involved in the UDN evaluation. The pediatric undiagnosed focus group underscored the importance of being able to rule out information and a new ability to come to terms with not having answers. We also discovered that both undiagnosed focus groups explicitly wanted ongoing communication and follow-up with the UDN over time to work towards a diagnosis.

Notably, previous research has described certain benefits of diagnostic evaluation and genomic testing for pediatric rare and undiagnosed populations only [7, 15, 37, 39, 40]. In this study, adult undiagnosed and diagnosed focus groups also highlighted the importance of UDN evaluation in validating the patient experience, providing access to referrals and providers, and incorporating ongoing follow-up communication as part of its process. As such, validation, access, and follow-up are likely crucial areas of focus for future research and practice.

### Future research

Further research must explore how systems can best facilitate the diagnostic journey for adults and children with rare and undiagnosed conditions. Specifically, work is needed to discern if multidisciplinary evaluations like those in the UDN fulfill an unmet need. Several study themes highlight that the current healthcare system is not set up to care for people with rare and undiagnosed conditions. In contrast, the UDN was noted to be comprehensive, in contrast to clinical encounters. Moreover, participants in this study talked about their gratitude for the UDN and wanted ongoing follow-up with the network. Currently, the UDN does not offer follow-up care. Further research is needed to explore whether providing clear and regular follow-up, including information communication alone (e.g., an email describing if new findings have or have not been seen in the last year based on genome sequencing), may benefit patient care in the long term. Thus, to improve systems navigation and care experience, and hopefully outcomes, we must more fully understand the role of these and other relevant components in the patient and caregiver experience.

In addition, formal measurement of the concept of future orientation in patients and families impacted by rare and undiagnosed conditions could help identify factors that influence outlook, and particularly the role of multidisciplinary evaluations. Further work could explore the impact of such evaluations on school and family relationships and career choices as well; two key themes that came from this study. Prior research has identified significant differences between UDN evaluations and clinical genomic services [24]. Therefore, another area of needed study is to determine whether themes noted here may also be relevant to clinical genomic services alone.

### Limitations

This study had several limitations. First, we only included individuals who spoke English due to funding and time constraints. For these reasons, additional focus groups were also not possible, but would have been preferred to increase sample size or include other perspectives (e.g., caregivers of adult participants). In addition, we limited focus groups to 60 min due to the Zoom context; nonetheless, we obtained saturation for all questions, as demonstrated by frequent answering of questions before they were asked, or within the time allotment for that question. Given that these sessions were conducted virtually, other individuals may have been present in the room. This or situations like it could have impacted focus group responses and willingness to share. Finally, a now-adult pediatric participant was involved in the adult undiagnosed focus group. At the time of the focus group, she was an adult and contributed from the perspective of managing her own care, though when she was evaluated in the UDN, she was a minor. Such limitations may reduce the generalizability of study findings.

## Conclusions

Rapid advances in science and medicine are changing care for patients with rare and undiagnosed conditions. We are moving quickly towards streamlined diagnosis and personalized treatment that will impact patient and family lives. It is therefore important to understand patient and caregiver perspectives in the implementation of advanced diagnostic approaches in both clinical and research settings. Our study findings are consistent with prior literature in the field focused on the patient experience of rare and undiagnosed conditions and highlight the various, related benefits from comprehensive evaluations, regardless of whether a diagnosis is obtained. Discussions with this group of UDN participants and caregivers also suggest areas for improvement and additional research related to the diagnostic odyssey.

## Data Availability

The focus group guide is available as Supporting Information. The transcribed focus group sessions used in this study are not publicly available. This is to protect and maintain participants' anonymity and confidentiality.
